# Suppression of NASH-Related HCC by Farnesyltransferase Inhibitor through Inhibition of Inflammation and Hypoxia-Inducible Factor-1α Expression

**DOI:** 10.3390/ijms241411546

**Published:** 2023-07-17

**Authors:** Kohei Yamada, Tomokazu Tanaka, Keita Kai, Shohei Matsufuji, Kotaro Ito, Yoshihiko Kitajima, Tatsuya Manabe, Hirokazu Noshiro

**Affiliations:** 1 Department of Surgery, Saga University Faculty of Medicine, Saga 849-8501, Japan; 19624021@edu.cc.saga-u.ac.jp (K.Y.); 19624016@edu.cc.saga-u.ac.jp (S.M.); sh2806@cc.saga-u.ac.jp (K.I.); kitajima.yoshihiko.eg@mail.hosp.go.jp (Y.K.); manabe@cc.saga-u.ac.jp (T.M.); noshiro@cc.saga-u.ac.jp (H.N.); 2 Department of Pathology, Saga University Faculty of Medicine, Saga 849-8501, Japan; kaikeit@cc.saga-u.ac.jp; 3 Department of Surgery, National Hospital Organization Higashisaga Hospital, Saga 849-0101, Japan

**Keywords:** non-alcoholic steatohepatitis, hepatocellular carcinoma, farnesyltransferase inhibitor, hypoxia-inducible factor-1α, anti-inflammatory response, nuclear factor-κB, transforming growth factor-β, Warburg effect, reactive oxygen species, interleukin-6

## Abstract

Inflammatory processes play major roles in carcinogenesis and the progression of hepatocellular carcinoma (HCC) derived from non-alcoholic steatohepatitis (NASH). But, there are no therapies for NASH-related HCC, especially focusing on these critical steps. Previous studies have reported that farnesyltransferase inhibitors (FTIs) have anti-inflammatory and anti-tumor effects. However, the influence of FTIs on NASH-related HCC has not been elucidated. In hepatoblastoma and HCC cell lines, HepG2, Hep3B, and Huh-7, we confirmed the expression of hypoxia-inducible factor (HIF)-1α, an accelerator of tumor aggressiveness and the inflammatory response. We established NASH-related HCC models under inflammation and free fatty acid burden and confirmed that HIF-1α expression was increased under both conditions. Tipifarnib, which is an FTI, strongly suppressed increased HIF-1α, inhibited cell proliferation, and induced apoptosis. Simultaneously, intracellular interleukin-6 as an inflammation marker was increased under both conditions and significantly suppressed by tipifarnib. Additionally, tipifarnib suppressed the expression of phosphorylated nuclear factor-κB and transforming growth factor-β. Finally, in a NASH-related HCC mouse model burdened with diethylnitrosamine and a high-fat diet, tipifarnib significantly reduced tumor nodule formation in association with decreased serum interleukin-6. In conclusion, tipifarnib has anti-tumor and anti-inflammatory effects in a NASH-related HCC model and may be a promising new agent to treat this disease.

## 1. Introduction

Obesity has become a prominent health concern worldwide as a risk factor for several chronic diseases, including diabetes mellitus, cardiovascular disease, and chronic kidney disease [1,2]. Furthermore, obesity is one of the most common and well-documented risk factors for non-alcoholic fatty liver disease (NAFLD) [3]. NAFLD is associated with a relatively low hepatocellular carcinoma (HCC) risk (annual incidence: 0.44 per 1000 person-years) [4]. However, the incidence of HCC increases up to 5.29 per 1000 person-years after the transition to non-alcoholic steatohepatitis (NASH) [4]. Vaccination and directly acting anti-viral agents have reduced the risk of virus-induced HCC [5,6]. However, there are few preventive strategies to stop NASH progression. Weight loss suppresses NASH-related HCC development, but it is difficult to achieve and sustain weight loss [7]. Therefore, another preventative or therapeutic strategy for NASH-related HCC is needed. NASH results from the pathological evolution of NAFLD following hepatic steatosis. Inflammation in NASH is mainly induced by several cytokines, such as interleukin (IL)-6 and tumor necrosis factor-α (TNF-α), produced by adipocytes and hepatic stellate cells, and gut-derived lipopolysaccharide (LPS) [8,9,10]. Although previous studies have revealed the effects of free fatty acids and cytokines on hepatocytes during NASH development [11,12,13,14,15], their pathological roles remain unclear in the progression of HCC from NASH.

Farnesyltransferase inhibitors (FTIs) suppress the proliferation of cancer cells, such as hematological or head and neck cancers [16,17,18,19]. Protein farnesylation is closely associated with inflammatory response and insulin signaling pathways in normal cells [20]. Hepatic steatosis is strongly associated with insulin resistance in the liver, and many antidiabetic drugs have been suggested to have a role in treating NAFLD and/or slowing down its progression to NASH and NASH-related HCC, as well as improving hyperglycemia in animal studies [21,22,23,24]. Thus, we hypothesized that protein farnesylation may be an important therapeutic target in NASH-related HCC, which is based on the chronic inflammation and insulin resistance of hepatocytes.

We and other researchers have shown that hypoxia-inducible factor (HIF)-1α promotes cancer aggressiveness through cancer cell proliferation, invasion, and metastasis [25,26,27,28]. HIF-1α is also a major mediator of inflammation that promotes cancer aggressiveness [29,30]. Previously, we demonstrated that 300 nM tipifarnib, which is an FTI, reduces HIF-1α expression in triple-negative breast cancer cells and suppresses migration, cancer stemness, and epithelial-to-mesenchymal transition regardless of RAS expression [31]. Additionally, we have shown that tipifarnib suppresses HIF-1α expression in gastric cancer cells and inhibits their proliferation and migration in vitro and in vivo [27]. Chronic inflammation of hepatocytes results in HCC development from NASH [32,33], but the mechanism remains unknown in relation to HIF-1α and FTIs.

In this study, we initially determined whether tipifarnib affected cell proliferation, metabolic changes, inflammation, and HIF-1α expression in human HCC cell lines cultured under several conditions of cytokines and free fatty acids mimicking the NASH environment. Then, we demonstrated multiple effects of tipifarnib on cancer progression and inflammatory response in a NASH-driven HCC mouse model induced by a high-fat diet and chemical carcinogen.

## 2. Results

### 2.1. HIF-1α Expression under Normoxia

**Figure 1:**
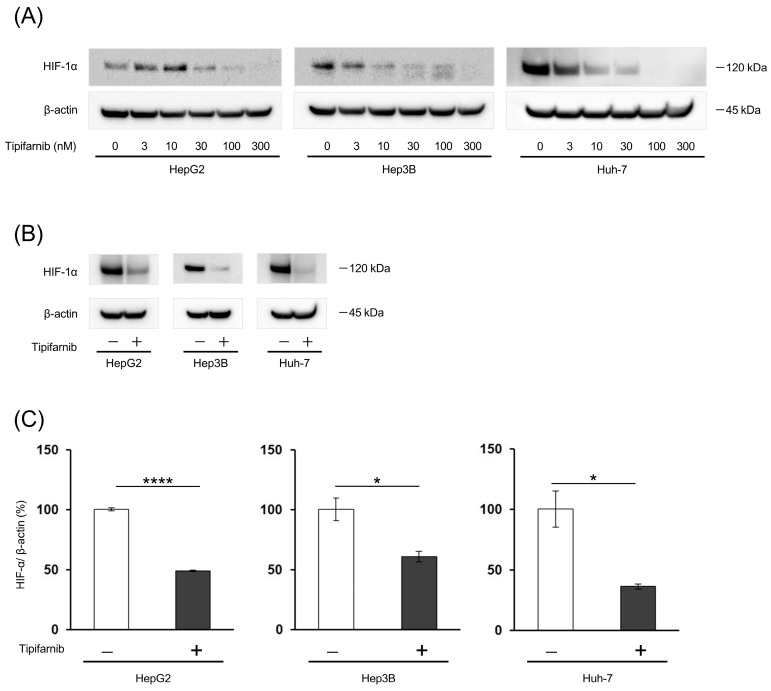
Effect of tipifarnib on hypoxia-inducible factor (HIF)-1α expression in HepG2, Hep3B, and Huh-7 cells. (**A**) HIF-1α protein expression was decreased in a dose-dependent manner by treatment with tipifarnib for 24 h. (**B**,**C**) HIF-1α expression was observed under normoxic conditions in these HCC cell lines. Treatment with 300 nM tipifarnib for 24 h significantly decreased the HIF-1α protein level. β-Actin as an internal reference was equally expressed in these cells. * *p* < 0.05, **** *p* < 0.0001 versus control. Unpaired *t* test.

HIF-1α expression was assessed in HepG2, Hep3B, and Huh-7 cells under normoxia. Tipifarnib decreased HIF-1α protein expression in a dose-dependent manner in these cell lines (Figure 1A). HIF-1α expression under 300 nM tipifarnib treatment was significantly decreased by 51.2% in HepG2 cells, 39.2% in Hep3B cells, and 63.6% in Huh-7 cells (Figure 1B,C). The most suppressive effect of tipifarnib was observed at 300 nM. Therefore, we used 300 nM tipifarnib in the following experiments.

### 2.2. HIF-1α Expression under NASH-like Conditions

**Figure 2:**
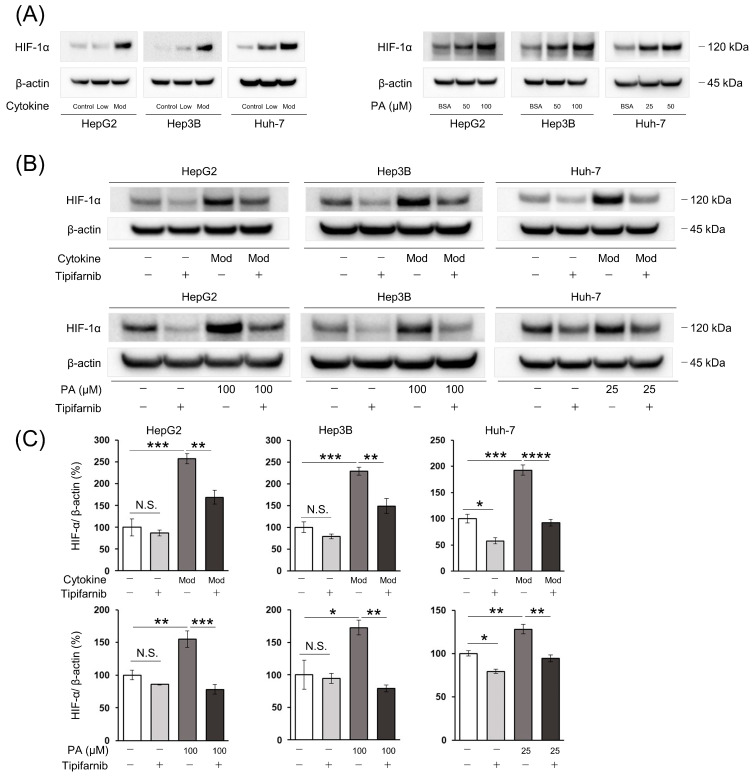
Effect of tipifarnib on HIF-1α expression under inflammation-induced and fatty-acid-loaded conditions in HepG2, Hep3B, and Huh-7 cells. (**A**) HIF-1α expression was increased by treatment with the cytokine cocktail (low and moderate concentrations) and palmitic acid (PA) (HepG2 and Hep3B cells: 50 and 100 μM; Huh-7 cells: 25 and 50 μM) for 24 h in a dose-dependent manner. (**B**,**C**) Treatment with tipifarnib (300 nM) for 24 h reversed the increased HIF-1α expression by treatment with the cytokine cocktail (moderate concentration) and PA (HepG2 and Hep3B cells: 100 μM; Huh-7 cells: 25 μM). Mod and Low indicate moderate and low concentrations of the cytokine cocktail, respectively. β-Actin as an internal reference was equally expressed in these cells. Expression under treatment with tipifarnib alone (gray bars) and the cytokine cocktail/PA without tipifarnib (dark gray bars) was compared with that of the control (white bars). Expression under treatment with the cytokine cocktail or PA with tipifarnib (black bars) was compared with that under treatment with the cytokine cocktail or PA without tipifarnib. * *p* < 0.05, ** *p* < 0.01, *** *p* < 0.001, **** *p* < 0.0001, N.S.: not significant. One-way ANOVA test followed by Tukey’s multiple comparison test.

In HepG2, Hep3B, and Huh-7 cells, treatment with low and moderate concentrations of the cytokine cocktail for 24 h significantly increased HIF-1α expression in a dose-dependent manner. Similarly, HIF-1α expression was increased by palmitic acid (PA) at 50 and 100 μM in HepG2 and Hep3B cells, and 25 and 50 μM in Huh-7 cells in a dose-dependent manner (Figure 2A). We also conducted the experiment with other concentrations, high and most high, of cytokine cocktail. Even these cytokine concentrations increased HIF-1α expression on all cell lines (Figure S1A). However, the cell viability assay demonstrated that the highest concentration (“most” high) of cytokine cocktail suppressed cell proliferation on HepG2 and Hep 3B for 72 h (Figure S1B). Therefore, we determined concentrations of cytokine cocktail as moderate and high for the following experiments. We basically also determined the 100 µM PA for the following experiments, based on HIF-1α expressions of each cell line. However, cell viability assay on Huh-7 with different concentrations of PA showed cell proliferation was exacerbated on more than 50 µM and cell death rate increased on more than 25 µM (Figure S1C). Therefore, we determined that the most optimal PA concentration for Huh-7 was 25 µM to avoid the emergence of cytotoxicity of PA itself. Tipifarnib strongly reversed the NASH condition-induced increase of HIF-1α protein expression in these cells (Figure 2B,C, S1A and S2).

### 2.3. Proliferation of HCC Cell Lines under NASH-like Conditions

**Figure 3:**
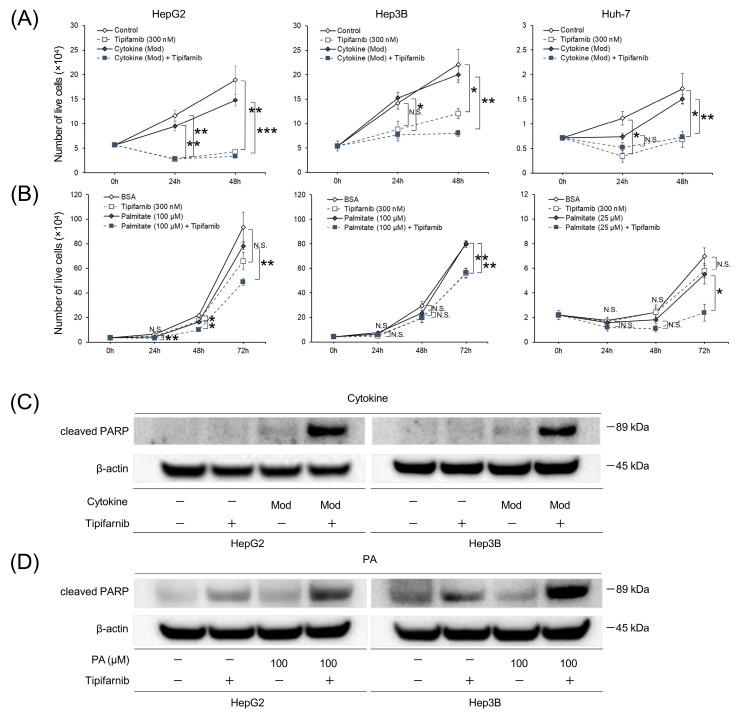
Effect of tipifarnib on cell proliferation and apoptosis under inflammation-induced and fatty-acid-loaded conditions in HepG2, Hep3B, and Huh-7 cells. (**A**) Numbers of live cells were assessed under the inflammation-induced condition (moderate concentrations of the cytokine cocktail) with or without 300 nM tipifarnib for 0–48 h. (**B**) Numbers of live cells were assessed under the fatty-acid-loaded condition (followed by the amount of PA) (HepG2 and Hep3B cells: 100 μM; Huh-7 cells: 25 μM) with or without 300 nM tipifarnib for 0–72 h. (**C**,**D**) Western blot analysis of cleaved poly(ADP-ribose) polymerase (PARP) expression was performed in HepG2 and Hep3B cells treated with moderate concentrations of the cytokine cocktail for 48 h (**C**) and 100 μM PA for 72 h (**D**) concurrently treated with or without 300 nM tipifarnib. β-Actin as the internal reference was equally expressed in these cells. * *p* < 0.05, ** *p* < 0.01, *** *p* < 0.001, control versus tipifarnib alone or cytokine cocktail/PA without tipifarnib versus conditions with tipifarnib, N.S.: not significant. One-way ANOVA test followed by Tukey’s multiple comparison test.

In the inflammation-induced model, trypan blue exclusion assays showed that tipifarnib significantly inhibited cell growth under treatment with or without moderate concentrations of the cytokine cocktail at 24–48 h in HepG2 cells and at 48 h in Hep3B and Huh-7 cells (Figure 3A). The cell death rate was significantly higher under treatment with the cytokine cocktail and tipifarnib than without tipifarnib at 24–48 h in HepG2 cells and at 48 h in Hep3B cells. Additionally, the cell death rate was significantly higher in the cytokine cocktail + tipifarnib group than in the tipifarnib alone group at 48 h in HepG2 and Hep3B cells (Figure S3A). Similarly, in the fatty-acid-loaded condition with PA at 100 μM in HepG2 and Hep3B cells and 25 mM in Huh-7 cells, treatment with tipifarnib significantly inhibited cell growth at 24–72 h in HepG2 cells and at 72 h in Hep3B and Huh-7 cells (Figure 3B). Cell death rates in the fatty-acid-loaded condition with tipifarnib were significantly higher than those without tipifarnib at 24–72 h in Huh-7 cells. Of note, the cytotoxic effect of tipifarnib on cell proliferation was significantly higher in the fatty-acid-loaded condition at 24 h in HepG2 cells and at 24–72 h in Huh-7 cells (Figure S3B).

### 2.4. Apoptosis of HCC Cell Lines under NASH-like Conditions

To further investigate the cytotoxic effect of tipifarnib, we examined the expression of cleaved poly(ADP-ribose) polymerase (PARP) in HepG2 and Hep3B cells cultured under the inflammatory condition for 48 h and the fatty-acid-loaded condition for 72 h in the presence or absence of 300 nM tipifarnib. Western blot analyses revealed cleaved PARP expression in HepG2 and Hep3B cells under the moderate concentrations of the cytokine cocktail + tipifarnib, but not tipifarnib alone for 48 h (Figure 3C). Similarly, cleaved PARP expression was observed under tipifarnib alone and 100 μM PA + tipifarnib for 72 h. However, cleaved PARP expression was significantly higher under treatment with PA + tipifarnib than with tipifarnib alone (Figure 3D). These results indicated that tipifarnib strongly induced apoptosis of HCC cell lines, and the apoptotic effect of tipifarnib was stronger under NASH-like conditions.

### 2.5. Glycolytic Metabolism in HCC Cells under NASH-like Conditions

**Figure 4:**
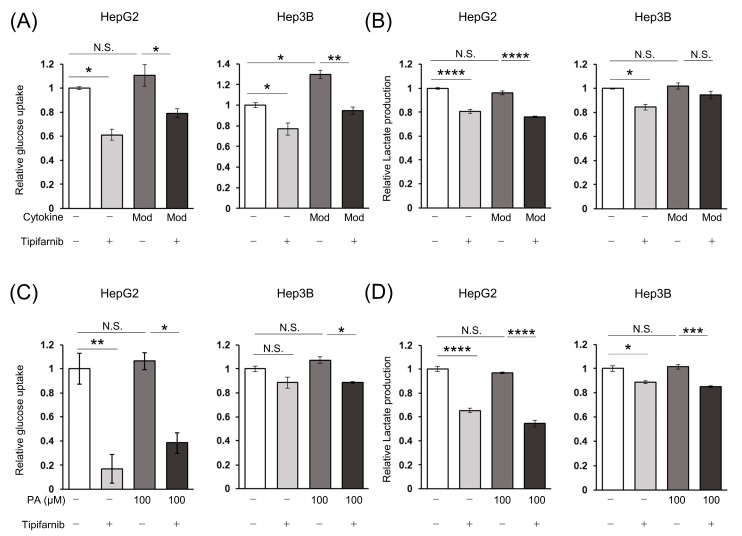
Effects of tipifarnib (300 nM) on glucose uptake and lactate production in HCC cells under non-alcoholic steatohepatitis (NASH)-like conditions. (**A**,**B**) Relative glucose uptake (**A**) and relative lactate production (**B**) were evaluated in HepG2 and Hep3B cells treated with moderate concentrations of the cytokine cocktail with or without tipifarnib for 48 h. (**C**,**D**) Relative glucose uptake (**C**) and lactate production (**D**) were evaluated under treatment with 100 μM PA with or without tipifarnib for 48 h. Control levels were set to 1. Levels under treatment with tipifarnib alone (gray bars) and the cytokine cocktail/PA without tipifarnib (dark gray bars) were compared with those in the control (white bars). Levels under treatment with the cytokine cocktail/PA with tipifarnib (black bars) were compared with those under treatment with the cytokine cocktail/PA without tipifarnib. * *p* < 0.05, ** *p* < 0.01, *** *p* < 0.001, **** *p* < 0.0001, N.S.: not significant. One-way ANOVA test followed by Tukey’s multiple comparison test.

To investigate whether HIF-1α expression decreased by tipifarnib-attenuated glycolytic metabolism, we evaluated the effects of tipifarnib on glucose uptake and lactate production in HepG2 and Hep3B cells. Glucose uptake was increased in both cell lines treated with the moderate concentrations of the cytokine cocktail as the inflammation-induced condition, particularly by approximately 1.3-fold in Hep3B cells. Tipifarnib significantly decreased glucose uptake even under the inflammation-induced condition and control (Figure 4A). Similarly, tipifarnib decreased lactate production under the inflammation-induced condition, although lactate production in both cell lines was not altered by treatment with the cytokine cocktail (Figure 4B). Glucose uptake by cells treated with 100 μM PA as the fatty-acid-loaded condition was also increased, but not significantly. Additionally, glucose uptake was significantly decreased by the addition of tipifarnib (Figure 4C). Lactate production was also significantly decreased by tipifarnib, whereas treatment with PA did not affect lactate production in HepG2 and Hep3B cells (Figure 4D). These findings suggested that tipifarnib attenuated glycolytic metabolism in hepatic cancer cell lines cultured under NASH-like conditions and HIF-1α played a major role in regulating this metabolic reprogramming.

### 2.6. Reactive Oxygen Species (ROS) Production in HCC Cells under NASH-like Conditions

**Figure 5:**
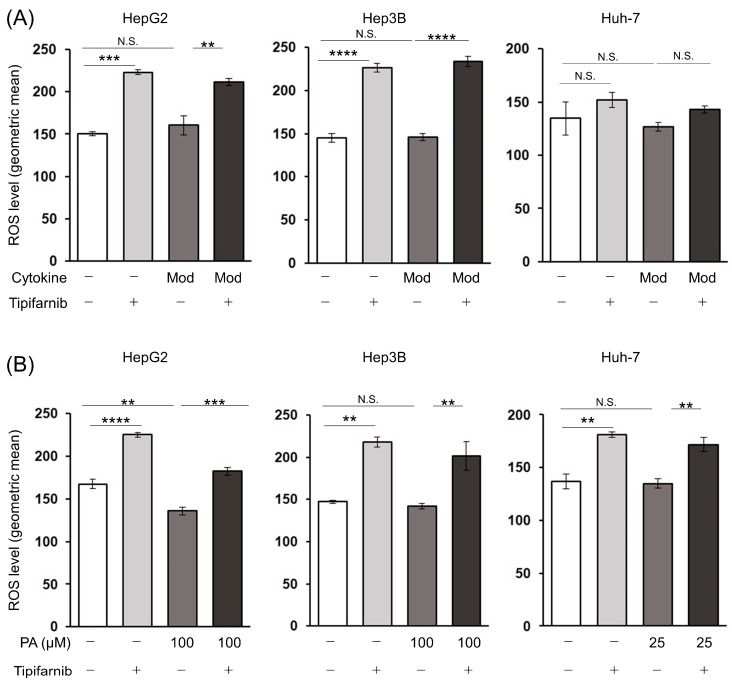
Tipifarnib increases intracellular reactive oxygen species (ROS) production in HepG2, Hep3B, and Huh-7 cells under NASH-like conditions. (**A**,**B**) Tipifarnib treatment (300 nM) for 48 h increased the intracellular ROS level in HCC cells treated with moderate concentrations of the cytokine cocktail (**A**) and 100 μM PA (**B**). Levels under treatment with tipifarnib alone (gray bars) and the cytokine cocktail/PA without tipifarnib (dark gray bars) were compared with those in the control (white bars). Levels under treatment with the cytokine cocktail/PA with tipifarnib (black bars) were compared with those under treatment with the cytokine cocktail/PA without tipifarnib. ** *p* < 0.01, *** *p* < 0.001, **** *p* < 0.0001, N.S.: not significant. One-way ANOVA test followed by Tukey’s multiple comparison test.

We next assessed ROS production underlying the induction of apoptotic cell death by tipifarnib in HepG2, Hep3B, and Huh-7 cells. Tipifarnib significantly increased ROS levels in HepG2 and Hep3B cells treated with or without the moderate concentrations of the cytokine cocktail. There was a trend of increased ROS levels by tipifarnib in Huh-7 cells regardless of the inflammation-induced condition, although this was not significant (Figure 5A). Similarly, tipifarnib significantly increased the ROS levels in HCC cells treated with or without 100 μM PA in HepG2, Hep3B, and Huh-7 cells. Neither the cytokine cocktail nor the PA affected the ROS level in the HCC cell lines, whereas the ROS level in HepG2 cells treated with only 100 μM PA was significantly decreased compared with that of the control (Figure 5B).

### 2.7. Intracellular IL-6 Level in HCC Cells

**Figure 6:**
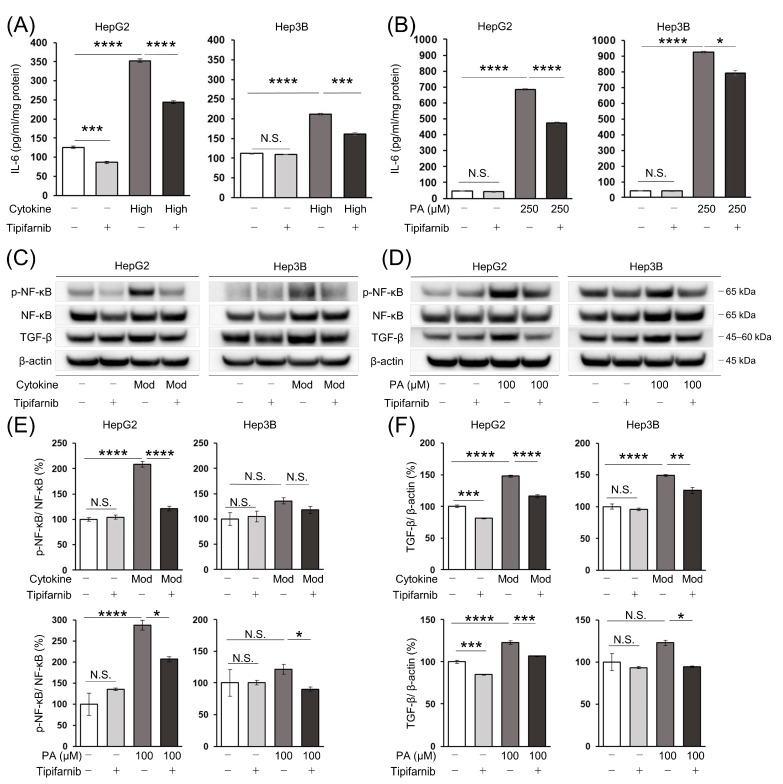
Tipifarnib reverses inflammation in HepG2 and Hep3B cells under NASH-like conditions. (**A**,**B**) Tipifarnib treatment (300 nM) for 24 h decreased the increase in intracellular interleukin (IL)-6 in HCC cells treated with high concentrations of the cytokine cocktail (**A**) and 250 μM PA (**B**). The IL-6 levels under treatment with tipifarnib alone (gray bars) and the cytokine cocktail/PA without tipifarnib (dark gray bars) were compared with that of the control (white bars). The level under treatment with the cytokine cocktail/PA with tipifarnib (black bars) was compared with that under treatment with the cytokine cocktail/PA without tipifarnib. (**C**,**D**) Nuclear factor-κB (NF-κB) and transforming growth factor-β (TGF-β) expression in HCC cells treated with moderate concentrations of the cytokine cocktail (**C**) and 100 μM PA (**D**) with or without tipifarnib (300 nM) for 24 h were detected by Western blotting. (**E**,**F**) Quantitative analysis of NF-κB (**E**) and TGF-β (**F**) protein expression. Levels under treatment with tipifarnib alone (gray bars) and the cytokine cocktail/PA without tipifarnib (dark gray bars) were compared with that of the control (white bars). Levels under treatment with the cytokine cocktail/PA with tipifarnib (black bars) were compared with that under treatment with the cytokine cocktail/PA without tipifarnib. * *p* < 0.05, ** *p* < 0.01, *** *p* < 0.001, **** *p* < 0.0001, N.S.: not significant. One-way ANOVA test followed by Tukey’s multiple comparison test.

To investigate the effects of FTIs other than ameliorating cancer cell proliferation and metabolism, we focused on the inflammatory response in the in vitro NASH-related HCC model. We measured the intracellular IL-6 level by an ELISA to determine whether tipifarnib improved the inflammatory response in HCC cells under NASH-like conditions. Even under the normal condition, tipifarnib significantly decreased the IL-6 level in HepG2 cells compared with that of the control, but not in Hep3B cells. The IL-6 level was significantly increased by the high concentrations of the cytokine cocktail in HepG2 and Hep3B cells. Tipifarnib significantly decreased the elevated level of IL-6, even under the inflammation-induced condition (Figure 6A). Similarly, the IL-6 level was significantly increased by 250 μM PA in HCC cells. Tipifarnib significantly suppressed the increased IL-6 level under the fatty-acid-loaded condition, similarly to the inflammation-induced condition (Figure 6B).

### 2.8. Nuclear Factor-κB (NF-κB) and Transforming Growth Factor-β (TGF-β) Protein Expression in HCC Cells

To further investigate the effects of tipifarnib on inflammation in HCC cells under NASH-like conditions, we evaluated the expression of NF-κB and TGF-β, a master mediator of closely linking inflammation and cancer [34,35]. The expression ratio of phosphorylated NF-κB, which was normalized to total NF-κB (p-NF-κB/NF-κB), was significantly increased in HepG2 cells treated with the moderate concentrations of the cytokine cocktail and 100 μM PA by 208% and 287% compared with control cells, respectively. Tipifarnib markedly inhibited the increase in the p-NF-κB/NF-κB ratio in these cells. There was a trend of the increased p-NF-κB/NF-κB ratio in Hep3B cells under inflammation-induced and fatty-acid-loaded conditions, although this was not significant. Tipifarnib decreased the p-NF-κB/NF-κB ratio in Hep3B cells under NASH-like conditions. However, significance was not reached under the inflammation-induced condition (Figure 6C–E). Simultaneously, the protein expression level of TGF-β was significantly increased in HepG2 cells under inflammation-induced and fatty-acid-loaded conditions. Tipifarnib treatment significantly inhibited the increase in TGF-β protein expression in HepG2 cells despite treatment with the cytokine cocktail or PA. TGF-β expression in Hep3B cells was markedly increased by the moderate concentrations of the cytokine cocktail. Although TGF-β expression in Hep3B cells was increased by 100 μM PA, it was not significant. Tipifarnib markedly decreased the increase in TGF-β expression in Hep3B cells treated with the cytokine cocktail or PA (Figure 6C,D,F).

### 2.9. NASH-Related HCC Mouse Model

**Figure 7:**
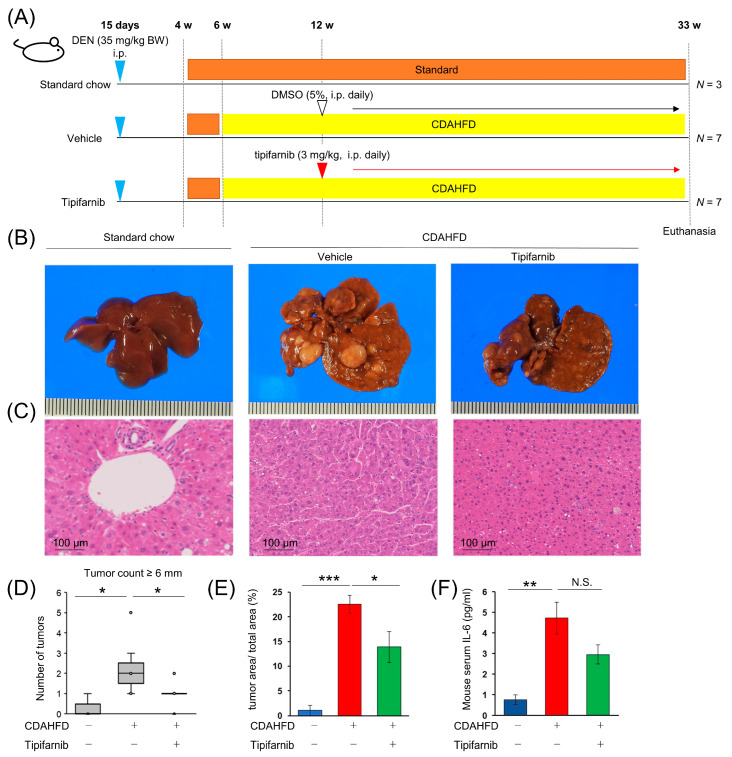
Tipifarnib alleviates HCC progression in the diethylnitrosamine (DEN) + choline-deficient, L-amino acid-defined, high-fat diet (CDAHFD) mouse model. (**A**) Experimental schema of the DEN + CDAHFD model using male C57/BL/6J mice. DEN (35 mg/kg) was injected intraperitoneally into 15-day-old mice (blue arrowhead). The mice were weaned at 4 weeks of age, followed by feeding on standard chow (brown bar) or the CDAHFD (yellow bar) after 6 weeks (w) of age for a total of 26 weeks. Either the vehicle (5% DMSO, intraperitoneal, daily; white arrowhead) or tipifarnib (3 mg/kg intraperitoneal, daily; red arrowhead) was administered starting at week 12 and ending at week 33 after birth. All mice were euthanized at week 33 to obtain liver and blood samples. (**B**) Representative macroscopic images of the liver surface. (**C**) Representative microscopy images of hematoxylin and eosin (H&E) staining of mouse livers. The image of standard chow-fed mice is normal liver parenchyma. Images of vehicle- and tipifarnib-treated mice show HCC. Scale bars have been added for reference. (**D**) Numbers of >6 mm macroscopic tumors were counted. (**E**) Ratio of the cross-sectional area of HCC/total liver was evaluated by a certified pathologist using whole scanned H&E-stained sections of the entire left liver. (**F**) Mouse serum levels of IL-6 were evaluated by an ELISA. Standard chow group (blue bar): *n* = 3 mice; CDAHFD with vehicle group (red bar): *n* = 7 mice; CDAHFD with tipifarnib group (green bar): *n* = 7 mice. * *p* < 0.05, ** *p* < 0.01, *** *p* < 0.001, N.S.: not significant. One-way ANOVA test followed by Tukey’s multiple comparison test.

We determined the effects of tipifarnib in the NASH-driven HCC mouse model. Figure 7A shows the experimental design of the diethylnitrosamine (DEN) + choline-deficient, L-amino acid-defined, high-fat diet (CDAHFD) mouse model. At week 33, CDAHFD + vehicle mice developed HCC with 100% incidence. No mice were excluded from or died in this study. Representative images of the liver in mice provided standard chow, CDAHFD + vehicle, or CDAHFD + tipifarnib are shown in Figure 7B. Histological examination showed that tumors had the characteristics of HCC: relatively small tumor cells with a high nuclear/cytoplasmic ratio, polymorphism of the nucleus, and coarse chromatin in hematoxylin and eosin staining. These characteristics were ameliorated in the tipifarnib group compared with the DMSO group (Figure 7C). On the liver surface, we macroscopically counted the number of tumors greater than 6 mm in diameter. Tipifarnib treatment significantly reduced the number of tumors (Figure 7D). Similarly, in the microscopic observation of the cut face of the liver, the area ratio of the tumor to the total liver was reduced by tipifarnib treatment (Figure 7E). To examine the inhibitory effect of tipifarnib on inflammation in vivo, we examined serum IL-6 levels by an ELISA. Compared with the standard chow group, the serum IL-6 level was significantly increased in the CDAHFD + vehicle group. The IL-6 level in the CDAHFD + tipifarnib group was reduced, but without significance (Figure 7F).

## 3. Discussion

We found that tipifarnib had both anti-tumor and anti-inflammatory effects in HCC cell lines in vitro and in the NASH-related HCC model in vivo. The protein level of HIF-1α was increased in HCC cells cultured under NASH-like conditions, and tipifarnib strongly reversed the expression of HIF-1α. The decrease in cell proliferation due to tipifarnib treatment was associated with HIF-1α expression. These findings suggest that HIF-1α plays a major role in the cancer aggressiveness of NASH-related HCC.

Hepatic steatosis and inflammation are essential for carcinogenesis in NASH patients and the aggressive phenotype of NASH-related HCC [36,37,38]. Therefore, we established inflammation-induced and fatty-acid-loaded in vitro conditions mimicking NASH. In HCC cell lines under NASH-like conditions, tipifarnib suppressed protein expression of NF-κB and TGF-β, which are associated with inflammation and fibrosis [39,40,41]. We also found that tipifarnib reduced the IL-6 level elevated by NASH-like conditions in vitro and in vivo. NF-κB regulates IL-6, a major inflammation-associated cytokine that evokes chronic inflammation in the liver [8]. The development of HCC associated with chronic inflammation requires NF-κB signaling in hepatocytes as an anti-apoptotic survival factor [42,43]. IL-6 mediates signal transducer and activator of transcription 3 (STAT3) activation, which promotes carcinogenesis in the liver. STAT3 is a major driver in the repair and replication of hepatocytes and enhances p300-mediated RelA acetylation, leading to nuclear retention of NF-κB. Thus, persistently activated STAT3 maintains constitutive NF-κB activity in hepatic tumor cells [44]. IL-6 and NF-κB maintain inflammatory responses in the liver, which are followed by the development and progression of HCC [34,41,45,46]. We consider that the mechanism of inhibited cancer aggressiveness by tipifarnib under NASH-like conditions may involve inhibition of the vicious cycle of inflammation-promoting cancer and cancer-promoting inflammation.

NASH progression begins by simple steatosis in hepatocytes. Subsequently, interactions between hepatocytes and immune cells, such as macrophages and fibroblasts, are crucial for the development of hepatitis, fibrosis, and HCC [47,48,49]. Therefore, the development of preventative therapies for NASH-related HCC requires in vitro cellular experiments and in vivo experiments using a NASH-related HCC animal model. Genetic and diet-related animal models are widely used to experimentally simulate the conditions of NASH-related HCC [50,51,52,53]. High-fat-diet-fed mice and genetic mouse models exhibit metabolic syndrome and severe steatosis, but not liver inflammation, fibrosis, or HCC in short-term experiments [54]. A methionine- and choline-deficient (MCD) diet and a CDAHFD both promote liver inflammation and fibrosis. However, the MCD diet model is difficult to apply in long-term experiments leading to carcinogenesis because of severe body weight loss [55]. The mouse model fed the CDAHFD is not related to the problem of weight loss, but this model requires at least 42 weeks to develop regenerative hyperplasia in the liver. Furthermore, the HCC incident rate in microscopic findings is only 27% at 66 weeks [56]. DEN, a highly reactive chemical carcinogen, is widely used to induce HCC in rodents [57]. The combination of CDAHFD feed and DEN injection strongly accelerates carcinogenesis and makes it possible to investigate therapeutic agents for NASH-driven HCC in a state more similar to the mild progression from hepatic steatosis, NAFLD, and NASH to HCC in a relatively short period [58]. In this study, tipifarnib was administered for 21 weeks, and we observed anti-tumor and anti-inflammatory effects. During the experimental period, significant body weight loss was not observed in the mouse groups treated with or without tipifarnib, and no mice died. These results have encouraged us to further investigate the clinical application of FTIs in humans with NASH and NASH-related HCC.

In this study, we did not conduct the in vitro experiments with a combination of the cytokine cocktail and PA treatment. Of course, instead of the “two-hit hypothesis” (first hit: hepatic steatosis, second hit: inflammation), which has insufficiently reflected the various molecular and metabolic pathways involved in NASH–HCC progression [59], the “multiple parallel hit hypothesis” has become recognized as the underlying pathophysiology of NASH and its progression to NASH–HCC, as the theory includes genetic, immunogenic, metabolic, and endocrine pathways [60]. The “multiple parallel hit hypothesis” has been involved in interactions between hepatocytes, sinusoidal endothelial cells, and immune cells, such as macrophages and lymphocytes [61]. We considered that this theory could be evaluated only in animal models, as adopted in many NASH and NASH–HCC preclinical studies. Furthermore, it is obvious that hepatic inflammation is one of the triggers of NASH. FTI is different from the previous drugs in terms of the multiple effects, which include not only anti-tumor effects but also anti-inflammatory and anti-diabetic effects. In the condition of combined cytokine and PA, cells might be affected from them simultaneously, and we could not evaluate the multiple effects of FTI clearly. Therefore, we only considered each condition of the cytokine cocktail or PA, respectively.

To determine whether the anti-tumor effects of tipifarnib were associated with HIF-1α, we focused on energy metabolism, particularly ROS production in cancer cells. ROS has dual contradictory activities in cancer development in accordance with its level: stimulation of tumorigenesis and cancer cell proliferation or induction of cell death [62]. HIF-1α controls ROS production under hypoxic conditions through multiple mechanisms, including the conversion of energy metabolism from oxidative phosphorylation to glycolysis, which is referred to as the Warburg effect [63,64,65,66]. We found that a low dose of tipifarnib suppresses the Warburg effect via HIF-1α in breast and gastric cancer cells under normoxia [27,31]. In the present study, we found that tipifarnib decreased lactate production and glucose consumption and simultaneously increased ROS production in response to suppressed HIF-1α expression under normoxia. Because tipifarnib induced apoptotic proteins, such as cleaved PARP, the excessive ROS production might be involved in the anti-tumor effect of tipifarnib.

Of note, tipifarnib exerted multiple effects in the NASH-related HCC models in vitro and in vivo. An anti-tumor effect was mediated via the suppression of HIF-1α, while the others were anti-inflammatory and anti-fibrotic effects, such as the downregulation of NF-κB, IL-6, and TGF-β. Regarding the anti-tumor effects of tipifarnib, we have reported that tipifarnib also exerts anti-tumor effects through the suppression of Rheb farnesylation, leading to the inhibition of the mTOR pathway other than via suppression of HIF-1α in gastric cancer cells [27]. In this respect, tipifarnib is speculated to have several mechanisms of anti-tumor effects, and it is superior to previous HIF-1α inhibitors, such as YC-1 and andrographolide [67,68]. Anti-inflammatory effects elicited by tipifarnib have been reported in acute liver failure and burn models in vitro and in vivo [20,69]. These previous reports strongly support our experimental results. Tipifarnib may have the specialized characteristic of simultaneously exerting anti-tumor and anti-inflammatory effects, which is rare for therapeutic agents of cancers. From the viewpoint of these multiple effects of tipifarnib, it may be the most suitable medicine regardless of the timing of administration for NASH-related HCC in which inflammation is deeply involved in its occurrence and development.

Some drugs have been researched for preventive effects for HCC. One of them is hydroxy-3-methyl-glutaryl-coenzyme A reductase inhibitors, referred to as “statins” [70]. Their increasing evidence shows that statins have anti-inflammatory and anti-oncogenic effects like tipifarnib [71,72]. Based on such multiple effects, statins have also been studied in NASH and NASH-driven HCC. These studies have showed that statins can reduce the risk of HCC occurrence not only with no disease background but also in NAFLD patients [70,73]. Although further study is needed, tipifarnib may also have preventive effects similar to statins for HCC in NASH patients.

In conclusion, tipifarnib is capable of downregulating HIF-1α expression in HCC cells cultured under NASH-like conditions. The tipifarnib-induced decrease in HIF-1α expression is associated with increased ROS production resulting in apoptotic cell death. Tipifarnib-induced decreases in NF-κB and TGF-β expression and the IL-6 level inhibit inflammation–cancer feedback. Thus, tipifarnib is a promising preventative and therapeutic agent for NASH-related HCC.

## 4. Materials and Methods

### 4.1. Cell Culture

Human hepatoblastoma cell line HepG2 and human HCC cell line Huh-7 were obtained from the Japanese Cancer Research Resources Bank (Osaka, Japan). Human HCC cell line Hep3B was obtained from the American Type Culture Collection (Manassas, VA, USA). All experiments were performed under normoxia (21% O_2_). The cells were cultured and maintained in 4.5 g/L glucose DMEM (Nacalai Tesque, Inc., Kyoto, Japan) supplemented with 10% heat-inactivated fetal bovine serum (FBS; Biowest, Nuaillé, France) and 100 mg/mL kanamycin (Meiji, Tokyo, Japan) at 37 °C and 5% CO_2_ in a humidified atmosphere.

### 4.2. Preparation of a Palmitic Acid (PA)/Bovine Serum Albumin (BSA) Complex Solution

A palmitic acid stock solution was prepared using a previously described method [74]. Briefly, a 100 mM solution of PA (P0500; Sigma-Aldrich, St. Louis, MO, USA) in 0.1 M NaOH solution (194-02191; FUJIFILM Wako Pure Chemical Corporation, Osaka, Japan) was heated at 70 °C in a shaking water bath. In an adjacent water bath, at 55 °C, a 10% (*w*/*v*) PA-free BSA solution (CultureSure, 034-25462; FUJIFILM Wako Pure Chemical Corporation) was prepared in ddH_2_O. A 5 mM PA/10% (*w*/*v*) BSA stock solution was prepared by adding 250 μL of the 100 mM palmitate solution dropwise to 4.75 mL of the 10% (*w*/*v*) BSA solution at 55 °C, followed by vortex mixing for 10 s and 10 min incubation at 55 °C. The PA/BSA complex solution was cooled to room temperature and sterile filtered (0.45 μm pore size membrane filter). At the same time, the PA-free BSA stock solution was prepared as a control. The complex solution was stored at −20 °C, where it was stable for 3–4 weeks. The stored 5 mM PA/10% BSA stock solutions were heated for 15 min at 55 °C and then cooled to room temperature before use.

### 4.3. Establishment of an In Vitro NASH-Related HCC Model

In vitro culture models of NASH-related HCC were established using a cytokine cocktail and PA. To induce an inflammatory response under hepatitis-like conditions, HepG2, Hep3B, and Huh-7 cells were treated with a cytokine cocktail of TNF-α, interferon-γ (IFN-γ), and LPS (T6674, SRP3058, and L4391, respectively; Sigma-Aldrich, St. Louis, MO, USA). The conditions of the cytokine cocktail were divided into three groups by the final concentrations, as follows: (1) most high concentrations (0.5 ng/mL TNF-α + 5.0 ng/mL IFN-γ + 1.0 mg/mL LPS); (2) high concentrations (0.25 ng/mL TNF-α + 2.5 ng/mL IFN-γ + 0.5 mg/mL LPS); (3) moderate concentrations (0.1 ng/mL TNF-α +1.0 ng/mL IFN-γ + 0.1 mg/mL LPS); and (4) low concentrations (0.05 ng/mL TNF-α + 0.5 ng/mL IFN-γ + 0.05 mg/mL LPS). To establish a fat-loaded condition mimicking steatosis, HCC cells were exposed to 25, 50, 100, and 250 µM PA. To determine the effect of tipifarnib (R115777; Selleckchem, Houston, TX, USA) in the in vitro NASH-related HCC models, HCC cells were treated with the cytokine cocktail or PA at several concentrations with or without 300 nM tipifarnib for 24, 48, and 72 h. Tipifarnib was administrated at the same time as the treatment of conditioned medium with the cytokine cocktail or PA simultaneously.

### 4.4. Western Blotting

Whole-cell lysates from cultured cells were prepared using lysis buffer composed of 150 nM NaCl, 50 mM Tris-HCl (pH 7.5), 2 mM EDTA, 1% Triton X-100, 1% sodium deoxycholate, 2% sodium dodecyl sulfate, 28 mM phenylmethylsulfonyl fluoride, and a protease inhibitor cocktail mix (Roche, Mannheim, Germany). Aliquots containing 30 mg of protein were electrophoretically separated in 5–20% Bis-Tris gels (Inter-Techno Co., Ltd., Tokyo, Japan) and transferred to Amersham Hybond P PVDF 0.45 membranes (Cytiva, Tokyo, Japan). Membranes were blocked with 5% skimmed milk at room temperature for 1 h and then incubated overnight at 4 °C with the indicated primary antibodies: anti-HIF-1α (1:1000 dilution, 610958; BD Biosciences, Franklin Lakes, NJ, USA), anti-PARP (1:1000 dilution, 5625S; Cell Signaling Technology, Danvers, MA, USA), anti-NF-κB (1:1000 dilution, 8242; Cell Signaling Technology), anti-phospho-NF-κB (1:1000 dilution, 3033S; Cell Signaling Technology), anti-TGF-β (1:1000 dilution, 3709S; Cell Signaling Technology), and anti-β-actin (1:10,000 dilution, AC15; Sigma-Aldrich). Following incubation with the corresponding secondary antibodies, the signals were developed using ECL Prime Western Blotting Detection Reagent (Cytiva, Tokyo, Japan). Images were acquired using a FUSION FX7.EDGE imaging system (Vilber Bio Imaging, Marne-la-Vallée, France). Densitometry was performed using Evolution-Capt software v17.01 (Vilber Bio Imaging).

### 4.5. Cell Viability Assay

The effect of tipifarnib on cell proliferation was assessed by counting the number of viable cells by a trypan blue exclusion assay using a TC20™ Automated Cell Counter (Bio-Rad, Hercules, CA, USA).

### 4.6. Measurement of Glucose Uptake and Lactate Production

Glucose uptake and lactate production were measured by a Glucose Assay Kit-WST and Lactate Assay Kit-WST (Dojindo Laboratories, Kumamoto, Japan), respectively, in accordance with the manufacturer’s instructions. We initially seeded 5.0 × 10^4^ HepG2 and Hep3B cells onto 6-well culture plates.

### 4.7. Quantification of the Intracellular ROS Level by Flow Cytometry

Intracellular ROS levels were measured using a Total ROS Detection Kit (Enzo Life Sciences, Farmingdale, NY, USA) in accordance with the manufacturer’s instructions. ROS fluorescence was detected using a FACSVerse flow cytometer (Becton-Dickinson, Franklin Lakes, NJ, USA) and analyzed using FlowJo version 10.0 software (Becton-Dickinson, Franklin Lakes, NJ, USA). The mean fluorescence of ROS production was determined automatically and presented as the geometric mean.

### 4.8. Measurement of Intracellular IL-6 Production

Intracellular IL-6 was measured using a Human IL-6 ELISA Kit (RAB0307; Sigma-Aldrich) following the manufacturer’s protocol. The total cells were lysed and diluted to a protein concentration of 1 mg/mL with indicated diluent buffer before measurement.

### 4.9. Animal Experiments

Animal experimental protocols were approved by the Institutional Animal Care and Use Committee of Saga University (approval no. A2019-025-0, 12 December 2019). The animals were kept under specific-pathogen-free conditions maintained at 25 °C, with a relative humidity of 50%, and illuminated by a 12 h light–dark cycle. They were provided with normal or specific sterile food and autoclaved water ad libitum. The animal experiments were performed on two or three mice in each cage as the experimental unit. All interventions were carried out during the light cycle. The sample size was determined with power analysis and the HCC incidence rate of mice [58,75]. The mice were excluded from this study when significant body weight loss (≥20%), signs of immobility, ruffled fur, or an inability to eat were observed. The NASH-driven HCC mouse model was established by the method of Li et al. [58]. This mouse model develops HCC at 30 weeks of age under a NASH background [58]. Male 7-day-old C57BL/6J mice were obtained from CLEA Japan, Inc. (Tokyo, Japan). A dose of 35 mg/kg diethylnitrosamine (DEN; N0258; Sigma-Aldrich) was injected intraperitoneally into the mice at day 15. At 6 weeks of age, mice were provided either standard chow (*n* = 3) or a choline-deficient, L-amino-acid-defined, high-fat diet (CDAHFD) (60 kcal% fat and 0.1% methionine by weight, A06071302; Research Diets, Inc., New Brunswick, NJ, USA) (*n* = 14) for 26 weeks. After 6 weeks of the CDAHFD, the mice were treated with daily intraperitoneal injections of 3 mg/kg/day tipifarnib (*n* = 7) or the vehicle [5% DMSO in 0.1 mL normal saline, *n* = 7] for 21 weeks. The mice were anesthetized for sacrifice. Terminal blood collection was performed by cardiac puncture. The number of liver tumors visible on the surface was counted macroscopically. Then, the left liver lobe was fixed in 3.5% formaldehyde, and the right lobe was snap frozen for further analysis.

### 4.10. Liver Histological Evaluation

Formaldehyde-fixed samples were examined histologically by hematoxylin and eosin staining. The area of HCC in the total liver of each microscopy image was marked by a certified pathologist blinded to the mouse characteristics. The ratio of the cross-sectional area of HCC to the total liver was calculated using NDP.view2 software (U12388-01; Hamamatsu Photonics, Shizuoka, Japan).

### 4.11. Measurement of Mouse Serum IL-6

The mouse serum level of IL-6 was measured using a mouse IL-6 ELISA Kit (RAB0308; Sigma-Aldrich) following the manufacturer’s protocol.

### 4.12. Statistical Analysis

Data were analyzed using JMP Pro version 16 (SAS Institute, Inc., Cary, NC, USA). The data were analyzed using the unpaired, two-tailed Student’s t-test when comparing two groups. To compare three or more groups, Tukey’s multiple comparisons test was performed for one-way ANOVA. *p* < 0.05 was considered statistically significant. All data are expressed as the mean ± SEM.

## Data Availability

All data generated or analyzed in this study are included in this paper and can be obtained from the authors upon reasonable requests.

## Supplementary Materials

The following supporting information can be downloaded at: https://www.mdpi.com/article/10.3390/ijms241411546/s1.
