# Supplementary material for: Suppression of NASH-Related HCC by Farnesyltransferase Inhibitor through Inhibition of Inflammation and Hypoxia-Inducible Factor-1α Expression
Source: Int J Mol Sci. 2023 Jul 17;24(14):11546. doi: 10.3390/ijms241411546 (PMC10380354; doi:10.3390/ijms241411546)
Supplement: Supplementary file 1 [file ijms-24-11546-s001.zip › ijms-2461766-supplementary.pdf]

Supplementary Material

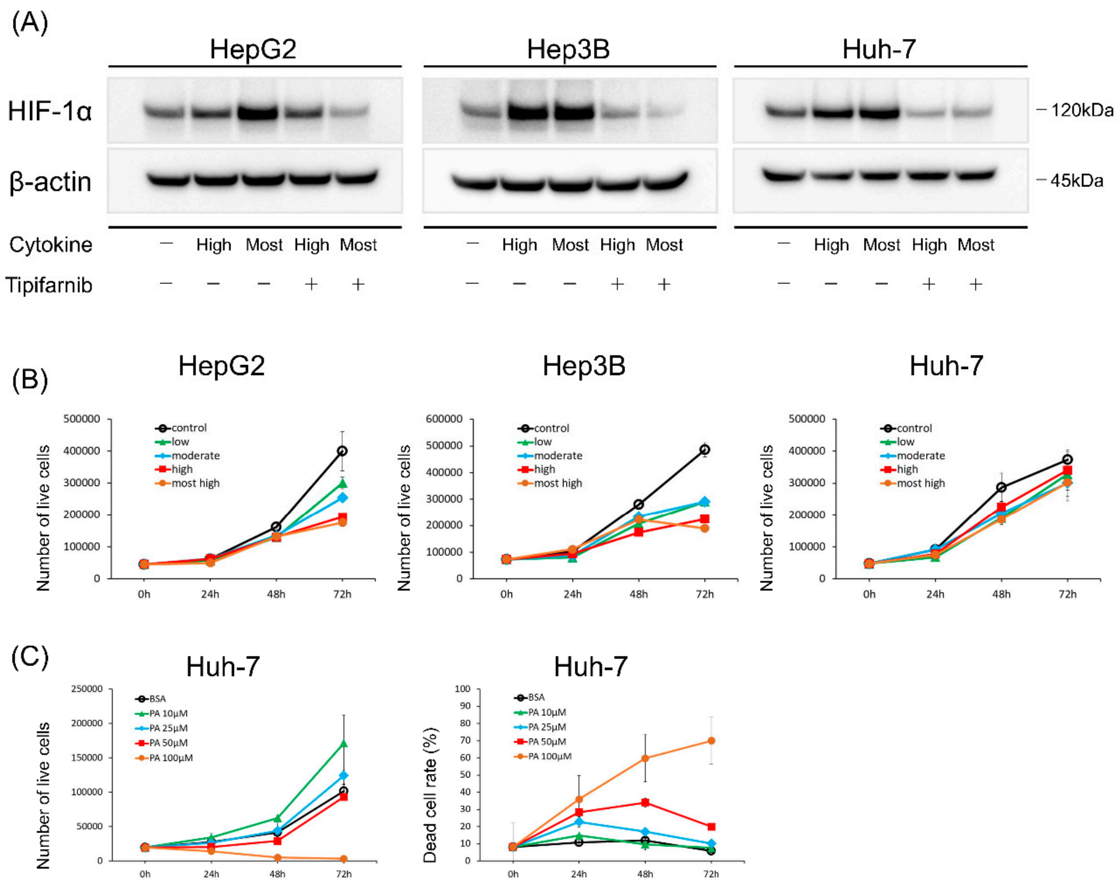

Figure S1. Western blot analysis showed the effect of tipifarnib on HIF-1α expression in all cell lines treated with high and the highest ("most" high) concentrations as the inflammation-induced condition. (A) Cell viability assay in all cell lines with the different concentrations of cytokine cocktail (B) and in Huh-7 cells with different concentrations of PA. (C)

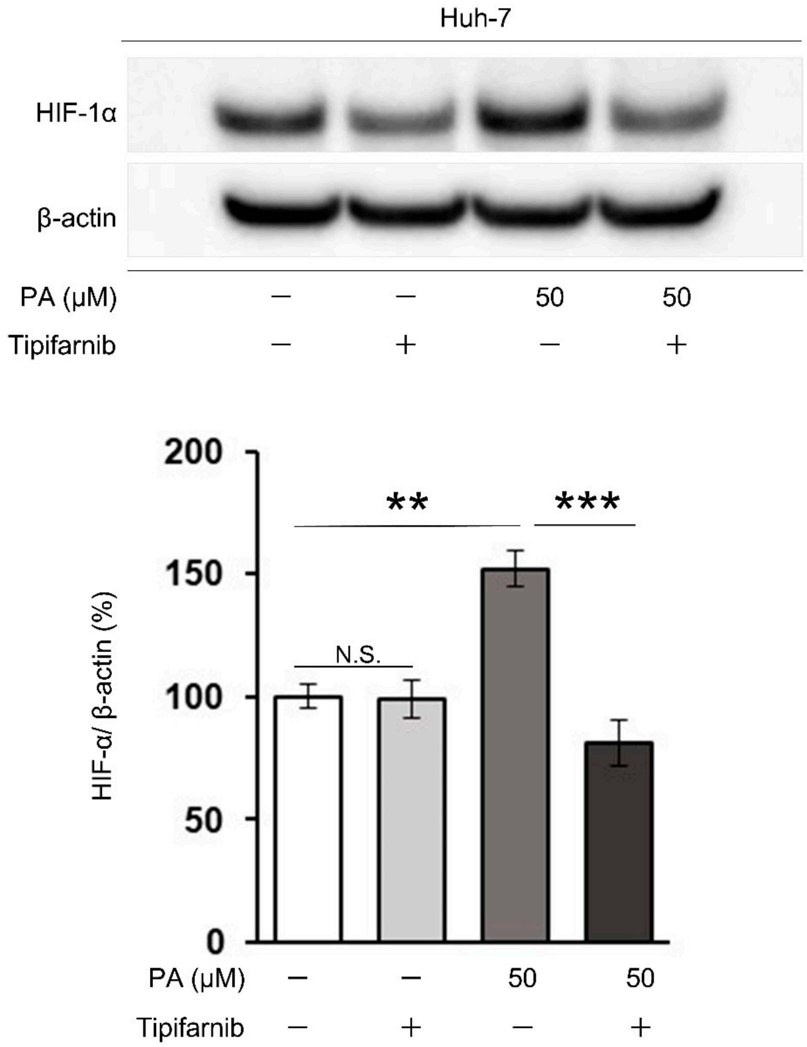

Figure S2. Effect of tipifarnib on HIF-1α expression in Huh-7 cells treated with 50 μM PA as the fatty acid-loaded condition. HIF-1α expression was increased by 50 μM PA treatment for 24 h similarly to 25 μM PA. Treatment with tipifarnib (300 nM) for 24 h reversed the increase in HIF-1α expression by PA treatment. **p < 0.01, ***p < 0.001, N.S.: not significant. one-way ANOVA test followed by Turkey's multiple comparison test.

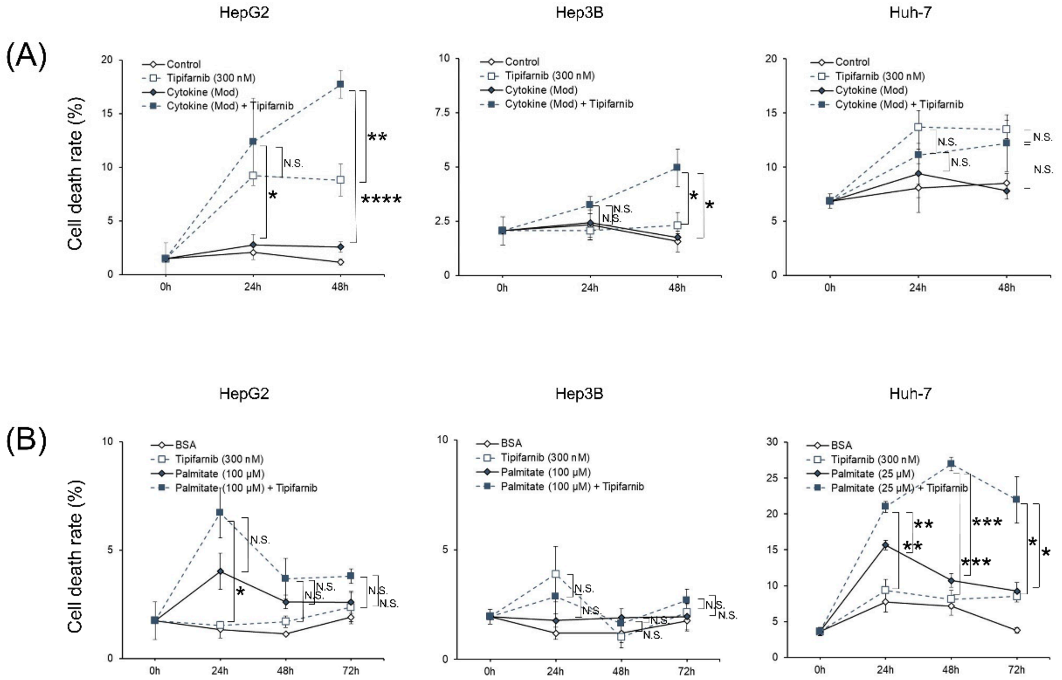

Figure S3. (A, B) Cell death was assessed under the inflammation-induced condition (moderate concentrations of the cytokine cocktail) for 0–48 h (A) and fatty acid-loaded condition (followed by the amount of PA) (HepG2 and Hep3B cells: 100 μM; Huh-7 cells: 25 μM) for 0–72 h (B) with or without 300 nM tipifarnib. Tipifarnib alone versus cytokine cocktail/PA with tipifarnib, cytokine cocktail/PA without tipifarnib versus conditions with tipifarnib. *$p < 0.05$, **$p < 0.01$, ***$p < 0.001$, ****$p < 0.0001$, N.S.: not significant. one-way ANOVA test followed by Turkey's multiple comparison test.
